# Emodin, a Natural Anthraquinone, Increases Uric Acid Excretion in Rats with Potassium Oxonate-Induced Hyperuricemia

**DOI:** 10.3390/ph16060789

**Published:** 2023-05-25

**Authors:** Shen-Wei Hou, Szu-Ju Chen, Jing-Dung Shen, Huey-Yi Chen, Shih-Jing Wang, Chia-Han Wang, Kee-Ming Man, Po-Len Liu, Ming-Yen Tsai, Yung-Hsiang Chen, Wen-Chi Chen

**Affiliations:** 1 Graduate Institute of Integrated Medicine, College of Chinese Medicine, China Medical University, Taichung 404, Taiwan; 2 Division of Urology, Department of Surgery, Taichung Veterans General Hospital, Taichung 407, Taiwan; 3 Division of Urology, Department of Surgery, Taichung Armed Forces General Hospital, Taichung 411, Taiwan; 4 National Defense Medical Center, Taipei 114, Taiwan; 5 Department of Obstetrics and Gynecology, Department of Medical Research, Department of Urology, China Medical University Hospital, Taichung 404, Taiwan; 6 Department of Chinese Medicine, Taichung Tzu Chi Hospital, Buddhist Tzu Chi Medical Foundation, Taichung 427, Taiwan; 7 Department of Medicinal Botanicals and Health Applications, College of Biotechnology and Bioresources, Da Yeh University, Changhua 515, Taiwan; 8 Department of Anesthesiology, China Medical University Hsinchu Hospital, Hsinchu 302, Taiwan; 9 Department of Respiratory Therapy, College of Medicine, Regenerative Medicine and Cell Therapy Research Center, Kaohsiung Medical University, Kaohsiung 807, Taiwan; 10 Department of Chinese Medicine, Kaohsiung Chang Gung Memorial Hospital and Chang Gung University College of Medicine, Kaohsiung 833, Taiwan; 11 Kaohsiung Municipal Feng Shan Hospital—Under the Management of Chang Gung Medical Foundation, Kaohsiung 830, Taiwan; 12 Department of Psychology, College of Medical and Health Science, Asia University, Taichung 413, Taiwan

**Keywords:** uric acid, gout, emodin, potassium oxonate, traditional Chinese medicine, rats

## Abstract

The treatment of hyperuricemia and gout is mostly based on lowering serum uric acid levels using drugs, such as allopurinol, or increasing urinary excretion of uric acid. However, some patients still experience adverse reactions to allopurinol and turn to Chinese medicine as an alternative. Therefore, it is crucial to design a preclinical study to obtain more convincing data on the treatment of hyperuricemia and gout with Chinese medicine. This study aimed to explore the therapeutic effect of emodin, a Chinese herbal extract, in a rat model of hyperuricemia and gout. In this study, we used 36 Sprague–Dawley rats, which were randomly divided into six groups for experimentation. Hyperuricemia was induced in rats by intraperitoneal injections of potassium oxonate. The efficacy of emodin in reducing serum uric acid levels was demonstrated by comparing the positive control group with groups treated with three different concentrations of emodin. The inflammatory profiles, including interleukin (IL)-1β, IL-6, and tumor necrosis factor-α levels, were unaffected by emodin treatment. In the experimental results, it was observed that the serum uric acid concentration in the vehicle control group was 1.80 ± 1.14, while the concentrations in the moderate and high concentration emodin groups were 1.18 ± 0.23 and 1.12 ± 0.57, resulting in no significant difference in uric acid concentration between these treatment groups and the control group, indicating that emodin has a therapeutic effect on hyperuricemia. The increase in the fractional excretion of uric acid (FEUA) demonstrated that emodin promoted urinary uric acid excretion without significantly affecting the inflammatory profile. Thus, emodin reduced the serum uric acid concentration to achieve effective treatment of hyperuricemia and gout by increasing urinary excretion. These results were supported by the measured serum uric acid and FEUA levels. Our data have potential implications for the treatment of gout and other types of hyperuricemia in clinical practice.

## 1. Introduction

Gout is a joint disorder caused by monosodium urate sediments [1,2]. It has been present in China since ancient times, with medical records reporting the disease among kings due to their purine-rich diet. Therefore, it is referred to as king’s disease. The incidence of gout has tended to increase because of an increase in the intake of refined foods and lifestyle changes [3]. Meanwhile, there is an association between diabetes, hypertension, cardiovascular disorders, obesity, and gout [4,5,6,7]. Therefore, the prevention and treatment of gout remain important issues.

Gout is characterized by increased serum uric acid levels and decreased urinary uric acid excretion. Hyperuricemia is an important risk factor for gout. The incidence of gout episodes increases when serum uric acid levels increase [8]. Primary gout is caused by genetic defects in purine metabolism enzymes, which increase uric acid production [9]. Factors involved in decreased urinary uric acid excretion include uric acid transporters in the gut and kidney, such as glucose transporter 9 (GLUT9; SLC2A9), urate transporter 1 (URAT1; SLC22A12), and ATP-dependent binding cassette transporter G family member 22 (ABCG22) [10]. Decreased urinary uric acid excretion increases serum uric acid levels [11]. Acquired gout is frequently associated with metabolic disorders, chronic kidney disease, and antineoplastic diseases [12]. The goal of treatment is to decrease the serum concentration or increase the urinary excretion of uric acid.

A review of herbs used in traditional Chinese medicine (TCM) showed that these herbs are effective at gout treatment by decreasing the production of uric acid, modulating uric acid transport, having anti-inflammatory effects, providing renal protection, and reducing free radical levels. However, some current herbal medicines used to treat gout have been observed to cause side effects. These side effects include diarrhea, nausea, indigestion, changes in the frequency of urination and defecation, increases in alanine transaminase levels, and leukopenia [13]. Emodin (1,3,8-trihydroxy-6-methylanthraquinone) is present in many TCM herbs, such as *Rheum palmatum*, *Polygonum cuspidatum*, *Polygonum multiflorum*, *Aloe vera*, and *Cassia obtusifolia*. Emodin has been used for the treatment of arthritis due to its anti-inflammatory effects, detoxification, and promotion of bowel movements. Emodin has also been reported to inhibit xanthine oxidase [14]. The activity of emodin and its derivatives is related to their effects on the inflammatory process and their immunomodulatory activity. Anti-inflammatory activity has been extensively studied in many disease entities. Emodin is an effective inhibitor of inflammatory markers such as nuclear factor (NF)-κB, tumor necrosis factor (TNF)-α, interleukin (IL)-1β, IL-6, IL-8, C-X-C chemokine receptor type 4 (CXCR4), and others [15]. Among them, NF-κB, TNF-α, IL-1β, IL-8 and other inflammatory markers have a close relationship with gout attacks. Therefore, emodin has potential for the treatment of gout.

In this study, we aimed to investigate the effects of emodin in the treatment of gout. The effective dosage, side effects, and liver toxicity were evaluated.

## 2. Results

### 2.1. Effect of Emodin on the Concentration of Serum and Urinary Uric Acid, Serum and Urinary Creatinine and FEUA in Hyperuricemic Rats

**Figure 1:**
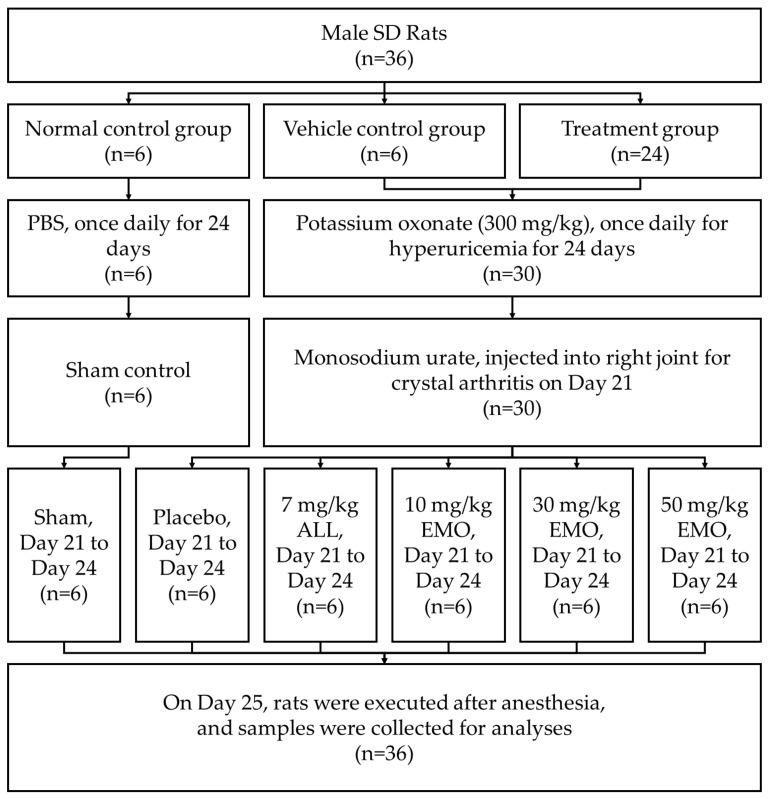
Flow diagram of the study of allopurinol and emodin on uric acid excretion in rats with potassium oxonate-induced hyperuricemia. SD: Sprague-Dawley, PBS: phosphate buffered saline, ALL: allopurinol, EMO: emodin.

The allopurinol (ALL) group was orally administered allopurinol after intraperitoneal (i.p.) administration. The EMO10, EMO30, and EMO50 groups (emodin 10, 30, and 50 mg/kg body weight, respectively) were administered emodin at the dosage described (Figure 1; Table 1).

The average serum uric acid concentration (SUA) was significantly higher in the VEH group than the control group 24 days after i.p. injection of potassium oxonate (PO) (Table 2, *p* < 0.05). These results indicated that the hyperuricemic rat model was successfully generated. The serum uric acid (SUA) was also significantly lower in the EMO30 and EMO50 groups than in the vehicle (VEH) group (*p* < 0.05). Although the SUA also decreased in the ALL group, the difference was not statistically significant. The urinary concentration of uric acid (UUA) and the fractional excretion of uric acid (FEUA) were significantly higher in the EMO30 and EMO50 groups than in the control group (*p* < 0.05). The results indicated that uric acid excretion was enhanced by moderate and high doses of emodin. The serum creatinine concentration was increased in the VEH group and significantly decreased in the EMO30 and EMO50 groups.

### 2.2. Serum and Urinary Cytokine Levels in Hyperuricemic Rats

There were no statistically significant differences in serum and urinary levels of interleukin (IL)-1β, IL-6, and tumor necrosis factor (TNF)-α between groups (Table 3). These results indicated that no inflammatory reaction was induced by PO in the rat hyperuricemic model. A significant difference in TNF-α levels was found between the EMO 30 and VEH groups (*p* < 0.05). However, there were no statistically significant differences between the EMO50 and VEH groups.

### 2.3. Changes in Liver Function and Serum and Urinary Urea Nitrogen Levels Induced by Emodin

There was a significant increase in liver function [serum glutamic-oxaloacetic transaminase (GOT) and glutamic pyruvic transaminase (GPT) levels] and serum and urinary urea nitrogen levels 24 days after PO injection in the study groups compared with the control group (Table 4, *p* < 0.05). This result indicated that PO caused liver and kidney injuries. Liver and renal function significantly improved after treatment with PO in the EMO10, EMO30, and EMO50 groups (*p* < 0.01). 

### 2.4. Changes in Xanthine Oxidase Activity in the Liver after PO Injection

**Figure 2:**
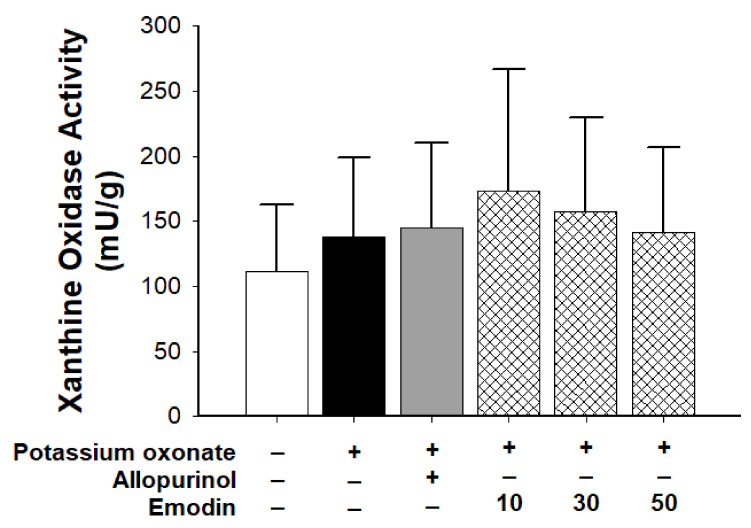
Xanthine oxidase activity in the control, vehicle, allopurinol (7 mg/body weight), and emodin groups (10, 30, and 50 mg/body weight, respectively). (*n* = 6 for each group; three independent experiments were performed in each group). Data are expressed as mean ± standard deviation (S.D.). One-way analysis of variance was performed.

Figure 2 shows the xanthine oxidase activity before and after PO injection. The activity did not change significantly between groups. Therefore, the effect of emodin on serum uric acid levels did not occur through the inhibition of xanthine oxidase.

### 2.5. Paw perimeter and Histopathology of Rat Fourth Intermetatarsal Space after Monosodium Urate Injection

**Figure 3:**
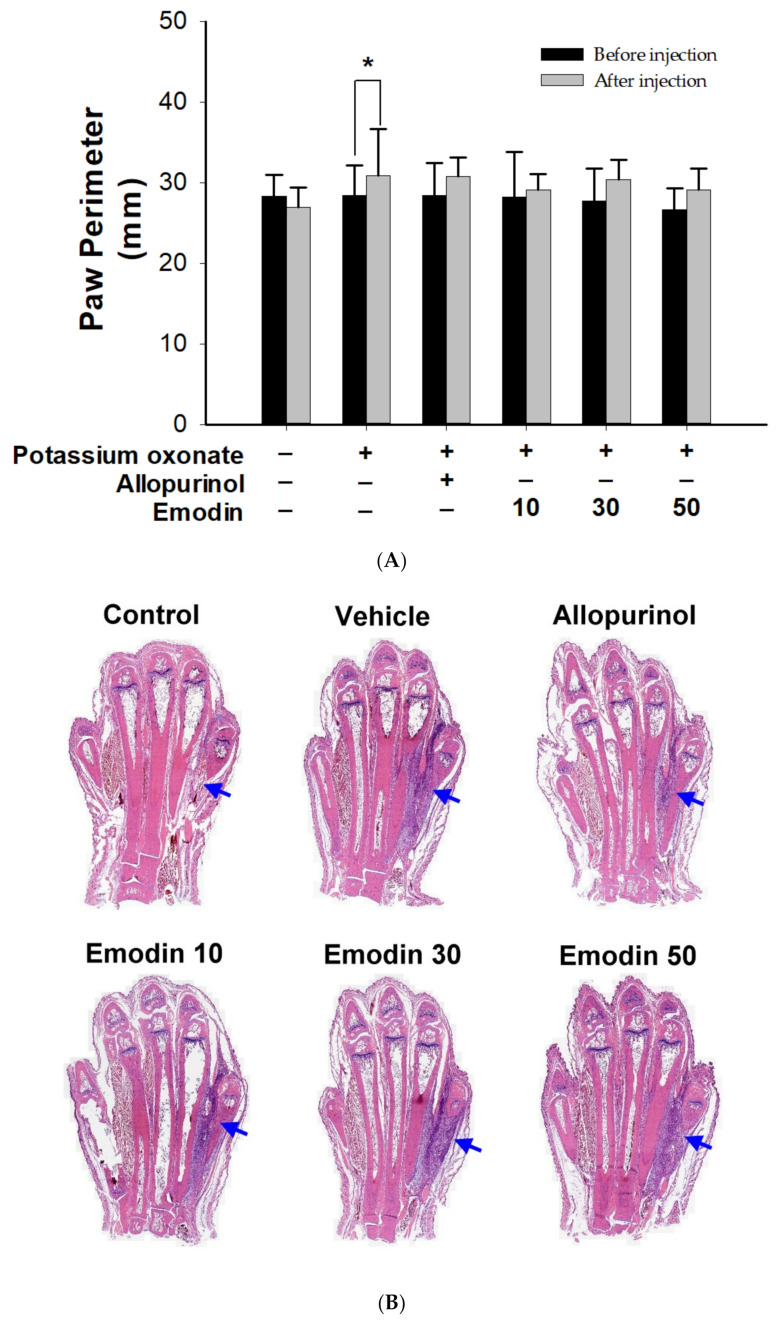
Paw perimeter and histopathology of rat’s fourth intermetatarsal space after monosodium urate injection. (**A**) Compared with the control group, all paws showed swelling after monosodium urate injection. There was no statistical difference between the emodin treatment groups and the VEH group. Data are expressed as mean ± S.D. One-way analysis of variance was performed. * *p* < 0.05 (**B**) Changes of rat’s ankle soft tissue between the 4th and 5th metatarsal bones (arrowhead) after local injection of monosodium urate. HE stain and TissueFAXS Viewer scan (20×). Allopurinol group, (7 mg/kg body weight); Emodin groups (10, 30, and 50 mg/kg body weight, respectively).

Compared with the control group, all paws showed swelling after monosodium urate injection (Figure 3A). There was no statistical difference between the emodin treatment groups and the VEH group. Emodin did not exert an anti-inflammatory effect on the acute phase of gouty joints.

After HE staining of the paw, the specimens were observed under a microscope and scanned using TissueFAXS (Figure 3B). No tissue swelling was observed in the control group. In contrast, tissue swelling of the 4th and 5th metatarsal bones was observed in the VEH, ALL, and emodin treatment groups. These results revealed that allopurinol and emodin did not affect the acute phase of gouty arthritis.

### 2.6. Histopathology of the Kidney in Hyperuricemic Rats

**Figure 4:**
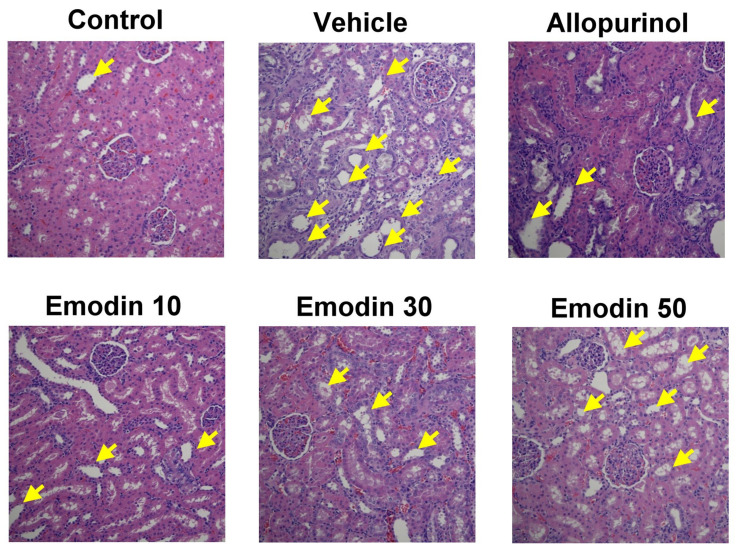
Representative paraffin sections of rat kidney histopathology viewed under a microscope (200×) in each treatment group [control, allopurinol (7 mg/kg body weight), and emodin groups (10, 30, and 50 mg/kg body weight, respectively)]. Sections were stained with hematoxylin and eosin and analyzed using TissueFAXS Viewer software. Compared to the control group, dilatation of renal tubules and inflammatory cell infiltration were observed in rat kidneys in the vehicle group. In the allopurinol group, there was also some infiltration of inflammatory cells, with less tubule dilatation. Conversely, in the emodin-treated groups, there was less dilatation and inflammatory cell infiltration in the renal tubules (arrowhead: dilatation of renal tubules and inflammatory cell infiltration).

Dilatation of renal tubules and inflammatory cell infiltration were observed in rat kidneys in the vehicle group (Figure 4). The results indicated renal tubule damage by PO-induced hyperuricemia. In the allopurinol group, there was also some infiltration of inflammatory cells, with less tubule dilatation. Allopurinol may partially reduce the effect of PO on tubule injury. There was less dilatation or inflammatory cell infiltration in the rat renal tubules in the emodin 10, 30, or 50 groups. These results indicated that emodin might have a renoprotective effect.

## 3. Discussion

We successfully developed an animal model of hyperuricemia in rats. PO significantly increased the serum level of uric acid 24 days after injection. However, emodin lowered serum uric acid levels and improved liver function, as indicated by GOT levels, in rats. A higher dose of emodin had a greater effect than allopurinol on lowering serum uric acid levels in this study. We also found that the therapeutic effects of emodin may be dose-dependent. However, emodin treatment had no effect on xanthine oxidase activity. Therefore, it was not possible to determine whether emodin inhibited xanthine oxidase activity.

**Figure 5:**
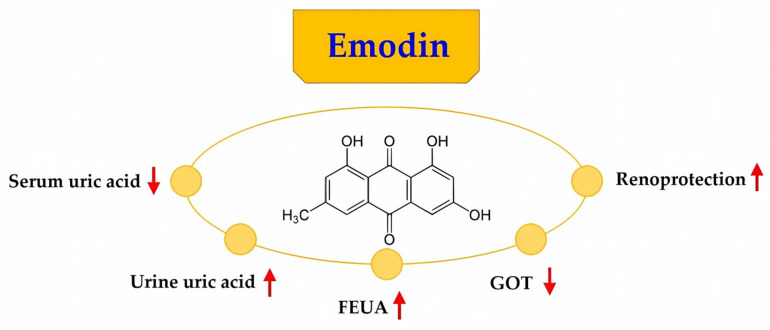
The possible collective diagram that demonstrates the entire results of emodin. FEUA: fractional excretion of uric acid, GOT: glutamic oxaloacetic transaminase.

Our data showed that medium and high concentrations of emodin increased FEUA. These results indicated that medium and high concentrations of emodin promoted uric acid excretion. Follow-up studies should aim to observe the changes in the protein and mRNA levels of factors involved in renal uric acid excretion to determine the mechanism whereby emodin promotes uric acid excretion in the kidney. Recent studies have shown that hyperuricemia is a risk factor for kidney disease and has a significant impact on the development of kidney disease and subsequent derivative diseases. One of the causes of hyperuricemia is an insufficiency of the renal uric acid transport system. Therefore, the regulation of the renal uric acid transport system may be a mechanism of action of emodin in the treatment of hyperuricemia. The possible collective diagram (Figure 5) that demonstrates the entire result of emodin.

Gout is a commonly observed, curable form of inflammatory arthritis. The precipitation of monosodium urate in the joints due to high serum uric acid levels is the major cause of gout [8,16]. In this study, there was no significant reduction in serum uric acid levels in any of the groups. The dose of allopurinol used in this experiment was based on the results of previous studies. The inability to significantly reduce uric acid levels may have been due to the low drug concentration or the poor solubility of allopurinol, resulting in insufficient doses administered during tube feeding. Subsequent experiments may consider increasing the dosage of allopurinol or using other administration methods.

With improvements in the quality of life, the prevalence of hyperuricemia and gout has increased annually [3]. Effective drugs are available for the clinical treatment of gout and hyperuricemia; however, some patients experience side effects, making treatment impossible and even life-threatening. Therefore, safe and effective drugs are urgently needed.

When the serum uric acid concentration is too high for a long time, deposition of uric acid crystals (monosodium urate) may occur. The deposited crystals then induce an acute inflammatory response known as a gout attack. More severe cases can result in tophi, chronic gouty arthritis (persistent joint inflammation caused by monosodium urate crystals), and structural joint damage. Gout attacks can occur in the joints or tissues surrounding the joints (e.g., synovial bursa, tendons, and soft tissue). Gout most commonly occurs in lower-extremity joints. Other locations, such as the distal knuckles, ears, olecranon sac, finger pads, and tendons (such as the Achilles tendon), may also be affected.

Monosodium urate, a component of tophi, is phagocytized by macrophages and other monocytes and induces an inflammatory response by activating the nucleotide-binding oligomerization domain-like receptor family pyrin domain-containing 3 (NLRP3) inflammasome. Activation of the NLRP3 inflammasome leads to the cleavage of pro-IL-1β and the secretion of IL-1β. IL-1β then binds to the IL-1β receptor, inducing the downstream signaling of pro-inflammatory cytokines and chemokines. Activation of the NALP-3 inflammasome causes monocytes and macrophages to release chemokines (e.g., CXCL-1, IL-8, and C-GSF), which promote neutrophil chemotaxis and proliferation. An increase in the number of macrophages and monocytes leads to the secretion of TNF-α and IL-1β, which cause a strong inflammatory response. The NLRP3 inflammasome does not directly induce an inflammatory response. Therefore, the deposition of monosodium urate crystals in the joints may not necessarily be accompanied by obvious inflammatory symptoms [17,18,19,20]. Since monosodium urate crystals tend to persist in the body for a long time, it is very important to control the inflammatory response and SUA levels. By understanding the pathogenesis and inflammatory mechanism of gout, we can control acute gout attacks, reduce SUA levels, and develop new drugs for its treatment. However, emodin did not exert anti-inflammatory effects in the present study.

Allopurinol is the first-line drug for the treatment of gout. Allopurinol is widely used clinically because of its availability, low cost, and therapeutic efficacy. It is a xanthine oxidase inhibitor that inhibits uric acid production. Allopurinol is well tolerated by most people; however, approximately 1–2% of users experience allergic reactions, such as a maculopapular rash. Although rare, patients treated with allopurinol may experience severe or even fatal skin reactions, such as Stevens–Johnson syndrome or toxic epidermal necrolysis [21]. Although the incidence rate is low, an adverse reaction to allopurinol is the most common cause of these disorders in Europe, Israel, and Taiwan [22]. Moreover, it is the second most common cause of drug rash with eosinophilia and systemic symptoms in Taiwan, Israel, and Europe. In addition, some patients experience other adverse drug reactions, such as acute kidney injury, hepatitis, and eosinophilia. This hypersensitivity reaction is genotype-dependent and most common in Southeast Asians and African Americans. Febuxostat is a xanthine oxidase inhibitor that inhibits uric acid production. However, febuxostat also has a risk of death and cardiovascular disease, and its mortality rate is higher than that of allopurinol [23].

Therefore, other compounds or natural Chinese herbal medicines can be used to replace or reduce the dosage of allopurinol to achieve the same or even greater therapeutic effects. Thus, the adverse reactions to allopurinol can be reduced with a reduction in its dosage. Chinese medicine has recorded hyperuricemia and gout for more than 2000 years. There is more than 1700 years of history of treating hyperuricemia and gout with Chinese herbal medicine, dating back to the synopsis of prescriptions in the Golden Chamber (Jingui Yaolue). Several Chinese herbal medicines have been used to treat gout and hyperuricemia in clinical practice [13,24,25]. Drugs containing emodin, such as rhubarb, aloe vera, cassia seed, knotweed, and other TCMs, should be safe for long-term human consumption [26,27]. However, to more accurately determine whether the drug is safe, this study also measured liver and kidney function. As a component of various edible plants, emodin has a good safety record for humans. Therefore, it is reasonable to consider the use of drugs containing emodin alone or in combination with existing drugs for the treatment of hyperuricemia and gout. Treatment with emodin-containing TCM may reduce or alleviate adverse reactions to allopurinol.

Our study was a pilot study of the effect of emodin on lowering serum uric acid concentrations by increasing urinary excretion of uric acid. Emodin is a chemical compound (6-methyl-1,3,8-trihydroxyanthraquinone) that is abundant in the TCM herb rhubarb. Recently, several studies have shown that emodin has anti-cancer, anti-bacterial, antiviral, antidiabetic, anti-inflammatory, anti-allergy, neuroprotective, liver-protective, and anti-osteoporosis activities [27]. However, the mechanism by which emodin excretes uric acid has hardly been studied. Nevertheless, we can still infer the possible mechanism of emodin based on the currently known methods of uric acid excretion and design subsequent experimental studies. Every day, 75% of uric acid salts are excreted through the kidneys. The excretion of uric acid by the kidneys involves four steps, including: (1) glomerular filtration; (2) pre-secretion reabsorption; (3) secretion; and (4) post-secretion reabsorption. Currently, the molecules controlling uric acid transport are still being studied. The excretion process relies on various uric acid salt transport proteins, including URAT1, organic anion transporters (OAT1 and OAT3), and adenosine triphosphate (ATP)-binding proteins, which are strongly correlated with uric acid salt excretion. The URAT1 transporter protein is regulated by the SLC22A12 gene, while GLUT9 is regulated by the SLC2A9 gene. In addition, uromodulin (UMOD) and Tamm-Horsfall glycoprotein are also related to uric acid excretion, but their mechanisms are still unclear [28]. In addition, an overdose (4000 mg/kg) or long-term use may lead to liver and kidney toxicity [29]. Our study used a relatively low dose of emodin, which demonstrated hepatoprotective and renoprotective effects. A high dose or long-term use of emodin should be considered in future studies.

This study had several limitations. We used rats as the animal model of hyperuricemia. Previously, a PO-induced hyperuricemia mouse model was constructed [30,31]. Due to the different animals used, we conducted a pilot experiment to confirm that hyperuricemia could be induced by PO before starting this study. When assessing the inflammatory pathway, our study was limited to the investigation of certain cytokines. It is not possible to identify all pathways. As this study was performed in rats, the findings may not fully represent the pathogenesis of hyperuricemia in humans [32,33,34]. 

In addition, the animal sample size used in our study may seem insufficient to yield conclusive findings. Nonetheless, we are committed to upholding ethical standards, and, as such, we cannot increase the number of animals at this time in line with the principles of replacement, reduction, and refinement. Despite the limited number of animals used, we were able to achieve statistically significant positive results in our study. In addition, a specified score for assessment of the histopathological changes in the renal tissues with immunohistochemical and electron microscopic examination of the renal tissues will add value to this manuscript. We unfortunately cannot perform this analysis due to various constraints such as time, budget, and technical expertise. However, we will explore the possibility of utilizing a specified scoring system to assess the histopathological changes in the renal tissues. 

The interaction with the microbiota is an important factor to consider in the study of emodin. It should be considered and explored for the potential impact of emodin on the microbiota fingerprint [35,36]. Additionally, we will examine the effects of emodin on the bioavailability of bioactive compounds and microbiota bioactivity in the near future. 

## 4. Materials and Methods

### 4.1. Chemicals and Reagents

Potassium oxonate (PO; high-performance liquid chromatography purity >97%, Product No.: 156124), carboxymethylcellulose (sodium salt, low viscosity, Product No.: C5678), Tween 80 (Product No.: P4780), uric acid (sodium salt, Product No.: U2875), dimethyl sulfoxide (DMSO, ≥99.9%, Product No.: 472301), and allopurinol (a xanthine oxidase inhibitor, Product No.: A8003) were purchased from Sigma–Aldrich (St. Louis, MO, USA). The uric acid inhibitory agent, emodin (90%, Product No.: ST-7788), was purchased from Combi-Blocks, Inc. (San Diego, CA, USA). All test agents were of analytical grade. Emodin (10 mg/kg body weight) was prepared by dissolving 10 mg in 0.05 mL of DMSO (≥99.9%) and adding water to a final volume of 10 mL (the final concentration of DMSO is 0.5%). Other doses of emodin were prepared as required. Serum and urinary biochemical profiles were assessed using commercial kits [37].

### 4.2. Animals and Treatment

Uric acid can be metabolized to allantois by uricase in animals. However, there is an evolutionary defect in uricase in humans. Animal models of gout are generated by intraperitoneal injection of PO, a uricase inhibitor. Gouty arthritis in rats was induced by an intra-articular injection of monosodium urate.

Six-week-old male Sprague–Dawley rats (*n* = 36) were purchased from BioLASCO Taiwan Co., Ltd. (Taipei, Taiwan). The rats were acclimated to the animal room for 1 week with a standard diet and ad libitum access to water. All protocols were approved (approval code: CMUIACUC-2021-393) by the Institutional Animal Care and Use Committee of the China Medical University (Taichung, Taiwan). Efforts were made to reduce the number of animals and their suffering. The rats were housed in pathogen-free conditions in an animal room with a humidity of 50 ± 10%, a 12 h/12 h light/dark cycle, and a temperature of 25 °C [38]. Before commencing the experiment, it is necessary to track the weight of the rats on a daily basis to ensure that their weight remains steady and that they are not exhibiting any signs of pain or discomfort. This will serve as an indicator that the rats have adjusted well to their surroundings. Once these conditions have been confirmed, the experiment can officially commence [39].

The rats were divided into the following groups (6 per group): (a) normal control, (b) hyperuricemia and gout (VEH), (c) allopurinol treatment (ALL, 7 mg/kg), (d) low-dose emodin 10 mg/kg (EMO10), (e) moderate-dose emodin 30 mg/kg (EMO30), and (f) high-dose emodin 50 mg/kg (EMO50) groups. The body weight of the rats was measured, and the rats were intraperitoneally (i.p.) injected every day from the start of the experiment until the end of the study (24 days). Phosphate-buffered saline (PBS; 10 mL/kg body weight, i.p.) was injected in rats in the control group, and PO (300 mg/kg body weight, i.p.) was injected as the induction agent in rats in the other groups. At day 21, the rats, except those in the control group, were anesthetized with 2.5% isoflurane and monosodium urate (0.1 mL, 120 mg/mL) into the right fourth intermetatarsal space. From the 21st day to the 24th day, oral gavage of 10 mL/kg bodyweight PBS was performed for the control and VEH groups. The ALL group was orally administered allopurinol after intraperitoneal administration. The EMO10, EMO30, and EMO50 groups were administered emodin at the dosage described above (Table 1). 

The perimeter of the paw was measured using silk and then transferred to a centimeter scale daily after injection. Urine samples (24 h) were collected from the metabolic cage the day before study completion, centrifuged at 2000× *g* for 5 min, and sent for analysis. On the 25th day, the rats underwent abdominal surgery under anesthesia. Blood samples were collected from the vena cava. The kidneys were excised during the operation. The right paw was cut for pathological examination. The rats were killed after the experiment was completed.

### 4.3. Biochemical Analyses

We measured uric acid, creatinine, and nitrogen levels in urine and blood samples. Blood samples were stored at room temperature for 1 h to allow coagulation. Serum was obtained after centrifugation of the blood samples (2500× *g*, 5 min). GOT, GPT, creatinine, and uric acid levels were measured using DiaSys GOT (ASAT) FS, DiaSys GPT (ALAT), DiaSys Creatinine FS, and DiaSys Urea FS (DiaSys Diagnostic Systems GmbH, Holzheim, Germany), respectively. TNF-α, IL-1β, and IL-6 levels were measured using enzyme-linked immunosorbent assay kits (LEGEND MAX™ Rat TNF-α ELISA Kit; BioLegend, Inc.; 438207, Rat IL-1β ELISA Kit; BioLegend, Inc.; 437107, LEGEND MAX™ Rat IL-6 ELISA Kit; Wuhan Fine Biotech Co., Ltd., Wuhan, China; ER1094). Paw specimens were examined using polarized microscopy to determine the presence of uric acid crystals. The minor lobe of the liver was homogenized in cold saline and centrifuged at 10,000× *g* for 10 min (4 °C). The supernatant was used to measure xanthine oxidase activity. Uric acid excretion was calculated as the fractional excretion of uric acid (FEUA; %), according to the following equation:

$$FEUA=\frac{urinary\;uric\;acid\times serum\;creatinine\;concentration}{serum\;uric\;acid\times urinary\;creatinine\;concentration}\times 100,$$

where the uric acid and creatinine concentrations are in mg/dL and mg/dL [40].

### 4.4. Histopathological Examination of Kidney

The process of preparing the kidney specimens for microscopic examination was carried out with utmost care and precision. The specimens were first fixed with formalin, a commonly used fixative that helps preserve the cellular structures of the tissues. Subsequently, the specimens were subjected to hematoxylin and eosin (H&E) staining, a widely employed staining technique that imparts color to the various components of the cells, thus facilitating their visualization under a microscope.

The stained tissue samples were then meticulously examined using TissueFAXS software, a powerful tool that allows for the acquisition of high-resolution images of biological samples. The use of this software was particularly advantageous as it enabled us to capture detailed images of the kidney tissues down to the subcellular level. By analyzing these images, we were able to discern subtle differences in the microstructure and cellular composition of the tissues, providing us with valuable insights into the biological processes occurring in these organs. Overall, this approach allowed us to obtain comprehensive and detailed microscopic results, which were critical to our research findings [41].

### 4.5. Outcome Assessment

The primary outcome was the serum uric acid concentration in rats. Swelling of the rat paw was evaluated on experimental days 21 and 25. The secondary outcome measures were the urinary uric acid concentration, serum and urinary creatinine concentrations, and serum biochemistry profile. The enzymatic activities of FEUA and xanthine oxidase were also evaluated. The urinary concentrations of the cytokines IL-1β, IL-6, TNF-α, and OSM were evaluated at the end of the experiment [42]. The histology of the kidney and paw bones was also evaluated.

### 4.6. Statistical Analyses

The data in this study were presented as the mean ± S.D. To perform the statistical analysis, the researchers utilized the Statistical Package for Social Sciences (SPSS 22.0; IBM, Armonk, NY, USA). To compare the differences between groups, a one-way analysis of variance was performed. The level of statistical significance was set at *p* < 0.05.

## 5. Conclusions

In this study, we successfully established a rat model of hyperuricemia. Emodin significantly lowered serum uric acid levels in rats with PO-induced hyperuricemia. Emodin also had renoprotective and hepatoprotective effects in rats with PO-induced hyperuricemia. This effect was attributed to increased uric acid excretion from the urine. Emodin had no anti-inflammatory effect on gouty arthritis. This requires further investigation to determine the pathway of renal excretion of uric acid. Overall, our findings indicate that emodin has clinical potential for the treatment of patients with hyperuricemia. In clinical use, emodin may be considered for independent use as a pure substance or in existing traditional Chinese herbs containing emodin to lower serum uric acid levels. This may help prevent or avoid the occurrence of gout, uric acid-induced kidney damage, and uric acid-related cardiovascular diseases.

## Figures and Tables

**Table 1:** Grouping of allopurinol and emodin treatments on hyperuricemic rats.

Groups	Treatments	Number of Rats	Dosage (mg/kg Body Weight)
Control	PBS + PBS	6	0
Vehicle	PO + PBS	6	0
Allopurinol	PO + ALL	6	7
Emodin (low)	PO + EMO	6	10
Emodin (medium)	PO + EMO	6	30
Emodin (high)	PO + EMO	6	50

PBS: phosphate buffered saline, PO: potassium oxonate, ALL: allopurinol, EMO: emodin. Total number of rats was 36.

**Table 2:** Biochemistry results of the rats.

	CON	VEH	ALL	EMO10	EMO30	EMO50
SUA (mg/dL)	1.16 ± 0.39 *	1.80 ± 1.14	1.30 ± 0.59	1.25 ± 0.75	1.18 ± 0.23 *	1.12 ± 0.57 *
SCr (mg/dL)	0.32 ± 0.82 *	0.40 ± 1.26	0.35 ± 1.67	0.33 ± 1.63	0.32 ± 0.82 *	0.32 ± 0.82 *
UUA (mg/dL)	5.72 ± 2.68 **	2.42 ± 0.75	2.10 ± 1.75	3.38 ± 2.18	4.60 ± 3.68 *	4.42 ± 3.84 *
UCr (mg/dL)	47.30 ± 24.51	36.31 ± 7.47	34.66 ± 7.72	36.33 ± 18.99	33.82 ± 12.98	36.67 ± 22.42
FEUA (%)	3.15 ± 1.12 **	1.61 ± 1.34	1.68 ± 1.55	3.14 ± 5.22	3.64 ± 2.09 **	3.72 ± 3.67 *

One-way analysis of variance was performed. * *p* < 0.05, ** *p* < 0.01. Compared with the VEH group. CON: control, VEH: vehicle (hyperuricemia and gout), ALL: allopurinol, EMO: emodin (mg/kg body weight), SUA: serum uric acid, SCr: serum creatinine, UUA: urine uric acid, UCr: urine creatinine, FEUA: fractional excretion of uric acid.

**Table 3:** Serum and urinary cytokine levels of rats.

	CON	VEH	ALL	EMO10	EMO30	EMO50
S IL-1β (pg/mL)	42.50 ± 55.14	40.10 ± 62.76	39.03 ± 58.44	33.88 ± 38.86	50.25 ± 56.96	64.80 ± 49.24
S IL-6 (pg/mL)	1.74 ± 2.00	2.01 ± 1.44	5.77 ± 17.46	2.68 ± 2.54	2.42 ± 2.44	2.11 ± 1.84
S TNF-α (pg/mL)	6.80 ± 4.66	7.03 ± 5.12	3.88 ± 4.48 *	7.55 ± 5.12	5.77 ± 3.62	5.42 ± 5.12
U IL-1β (pg/mL)	195.55 ± 89.18	168.27 ± 60.28	139.47 ± 31.76	149.02 ± 39.46	140.08 ± 60.80	171.32 ± 177.44
U IL-6 (pg/mL)	2.69 ± 2.36	2.11 ± 2.32	2.70 ± 3.20	3.43 ± 2.98	3.16 ± 1.86	1.95 ± 2.54
U TNF-α (pg/mL)	3.86 ± 2.20	4.44 ± 4.96	4.91 ± 4.38	5.42 ± 5.38	7.71 ± 3.84 *	6.86 ± 8.16

One-way analysis of variance was performed. * *p* < 0.05. Compared with the VEH group. CON: control, VEH: vehicle (hyperuricemia and gout), ALL: allopurinol, EMO: emodin (mg/kg body weight), S: serum, IL: interleukin, TNF: tumor necrosis factor, U: urine.

**Table 4:** Liver and renal function changes in the rats.

	CON	VEH	ALL	EMO10	EMO30	EMO50
GOT (U/L)	122.83 ± 54.00 *	163.00 ± 60.54	121.00 ± 27.68 *	99.67 ± 31.42 **	101.00 ± 48.92 **	84.67 ± 15.48 **
GPT (U/L)	39.17 ± 12.48	35.50 ± 19.62	36.50 ± 11.50	31.67 ± 12.94	30.83 ± 18.82	26.33 ± 10.48
S BUN (mg/dL)	18.50 ± 3.70 *	27.48 ± 13.42	25.25 ± 9.18	28.77 ± 20.14	24.63 ± 9.86	29.48 ± 12.36
U BUN (mg/dL)	10.77 ± 11.46 *	19.50 ± 14.06	13.93 ± 12.06	36.82 ± 140.60	57.03 ± 102.48	36.32 ± 102.46

One-way analysis of variance was performed. * *p* < 0.05, ** *p* < 0.01. Compared with the VEH group. CON: control, VEH: vehicle (hyperuricemia and gout), ALL: allopurinol, EMO: emodin (mg/kg body weight), GOT: glutamic oxaloacetic transaminase, GPT: glutamic pyruvic transaminase.

## Data Availability

The article contains original contributions to research, and further inquiries can be directed to the corresponding author.
